# Pros and cons of sociality across ecological niches: a swarm robotics approach

**DOI:** 10.3389/fpsyg.2026.1872416

**Published:** 2026-09-08

**Authors:** Roman Miletitch, Nicolas Cambier, Antonio Benítez-Burraco, Limor Raviv

**Affiliations:** 1 Language Evolution and Adaptation in Diverse Situations (LEADS) Group, Max Planck Institute for Psycholinguistics, Nijmegen, Netherlands; 2 Computational Intelligence Group, Vrije Universiteit Amsterdam, Amsterdam, Netherlands; 3 Department of Spanish, Linguistics and Theory of Literature (Linguistics), Faculty of Philology, University of Seville, Seville, Spain; 4 Donders Center for Cognition (DCC), Radboud University, Nijmegen, Netherlands

**Keywords:** cooperation, ecological conditions, fitness, foraging, collective decision-making, language evolution, sociality, swarm robotics

## Abstract

The evolution of language is closely linked to an increase in human sociality. But when is increased sociality an adaptive strategy? Swarm Robotics, which studies the collective behaviors of large populations of interacting robots with simple embodied cognition, is an ideal testbed for studying the environmental conditions that may favor the evolution of more social behaviors and cooperative language. Here, we introduce the notion of parametrizable sociality to classic swarm robotics models, and test the foraging and communication behaviors of robots with different degrees of sociality under different environmental conditions, which have been previously implicated in eliciting this increase in prosocial behavior in human history. Our results show that in abundant environments with plenty of resources, higher innate sociality is consistently beneficial only in the absence of aggressive competition. Nevertheless, social agents typically outperform aggressive agents in the short-term in both abundant and scarce environments. In scarce environments with fewer resources, this short-term advantage is especially beneficial as it enables agents to forage as much food as possible before depletion. Together, our results suggest that sociality provides an evolutionary advantage in specific conditions, which maps to the potential ecological niches in which early humans have evolved.

## 1 Introduction

Language is fundamentally social (Halliday, 1971; Schieffelin and Ochs, 1986). It depends on repeated interactions between individuals, on the motivation to engage with others, and on the ability to establish and maintain shared conventions over time. This deep link between sociality and language has been formulated from several perspectives, and is a prominent feature of any evolutionary theory concerning the origins of human language (Arnon et al., 2025). These include, for example, the “social brain hypothesis” (Dunbar, 1998; Oesch, 2024; Dunbar, 2003), which proposes that the cognitive demands of increasingly complex social groups shaped human cognitive evolution; the human “interaction engine” (Levinson, 2020; Heesen and Fröhlich, 2022), which emphasizes humans' strong motivation and ability to engage in coordinated social interaction; and the “human self-domestication hypothesis” (Hare, 2017; Thomas and Kirby, 2018; Beńıtez-Burraco, 2025), which suggests that reduced reactive aggression and increased prosociality created more favorable conditions for the emergence of complex culture and language. Across these approaches, language and sociality are not seen as independent traits. Rather, they are evolutionary and developmentally linked through a positive feedback loop: sociality creates more opportunities for communication, while successful communication in turn reinforces social bonds and increases the likelihood of future interactions.

Although high sociality (and consequentially, sophisticated communication) may seem like a fruitful evolutionary strategy, many animals live solitary lives or restrict their social bonds only to close kin. Notable exceptions are social insects and cooperatively breeding mammals, including non-human primates and humans. In these groups, social interactions can support coordinated behavior: honeybees recruit nestmates to food sources through waggle dances, while schooling fish and flocking birds coordinate their movements through local responses to nearby group members (Dyer, 2002; Katz et al., 2011; Ballerini et al., 2008). Given that increased sociality is not always favored by natural selection, as social interactions can also involve costs such as competition and conflict, the selection pressures and ecological conditions that favor the evolution of more social species remain a hot and ongoing topic of debate (Burkart, 2017; Le Galliard et al., 2005; Korb and Heinze, 2016; Schülke and Ostner, 2012).

For humans, many theories associate the evolution of our high sociality with species-internal pressures such as cultural group selection, reciprocity, emotional development, and punishment (Richerson and Boyd, 1998; Batson, 2012; Zahn-Waxler and Smith, 1992). However, much less work has focused on the ecological conditions that may have put humans on this evolutionary trajectory. Notably, different feeding habits and different resource distributions require different amounts of competition/cooperation. Illustrating this point, Batek women, who forage on location-specific and short-term variance resources (fishing points), have been found to maintain highly connected cooperative networks (Kraft et al., 2023). In contrast, Tsimane horticulture, which is based on farming distinct territories, typically involves more restricted and more modular social networks. The benefits of cooperation are likely to depend on the ecological context: for example, cooperation may be especially useful when resources are patchy, variable, or difficult to exploit alone (Pisor and Gurven, 2016). Since the ability to feed both oneself and one's kin is essential to propagating one's genome, current social theories suggest that early hominids have evolved in a specific ecological niche that required cooperation in order to feed. However, which environments promoted this increased sociality remains unclear. In this study, we use the term ecological niche to refer to the combination of environmental and social conditions that shape a population's opportunities for survival and reproduction. We focus specifically on resource availability, social conditions and spatial distribution, as these affect the costs and benefits of foraging, competition, and cooperation.

In fact, as it stands, selection toward more sociality in the human lineage has been linked to two very different environments. On one hand, abundant environments in which food supply is rich and reliable are thought to significantly relax the survival pressures that generally favor aggressive and competitive behaviors in nature, thus potentially giving room to more prosocial behaviors (Pisor and Surbeck, 2019; Hare et al., 2012). On the other hand, harsh environments with fewer resources and/or large spatial and temporal fluctuations in resource availability have also been thought to promote tolerance and cooperative behaviors, as they force the reliance on non-local food sources and resource sharing as means of managing the risks of resource shortfalls (Spikins et al., 2021). Yet direct evidence for either proposal is currently lacking. The goal of the current paper is to shed light on the potential ecological conditions that may favor sociality by simulating the impact of resource distribution and cooperation on the foraging and communicative behaviors of artificial agents, namely, swarm robots. In this article, the terms *robots* and *agents* are used interchangeably.

Swarm robotics is an approach to multi-agent systems inspired by social insects, which studies the emergence of complex collective behaviors such as foraging, aggregation, and communication as a result of numerous local interactions between relatively simple robots (Trianni et al., 2003; Brambilla et al., 2013; Reina et al., 2015). Foraging has been extensively studied in swarm robotics (Bonabeau et al., 1996; Krieger et al., 2000; Liu et al., 2007; Miletitch et al., 2018), and has already been combined with language games to show that an adaptive language can benefit swarm activities (Miletitch et al., 2022). This is part of a handful of studies that tested the mutual impact of language games and swarm behaviors (Trianni et al., 2016; Cambier et al., 2017, 2021), and demonstrated that agent behavior (Cambier et al., 2017), or even physical collisions, (Trianni et al., 2016) can create segmented social clusters, leading to slower convergence (Trianni et al., 2016) and spatially segregated sub-groups developing their own vocabulary (Cambier et al., 2017). Such segregation can prevent convergence toward a single vocabulary between sub-groups, allowing for a more exhaustive linguistic description of the environment (Miletitch et al., 2022). This spatial segregation can also allow robots to adapt their behavior to changing swarm conditions. In Cambier et al. (2021), robots propagated controller parameters that determined whether they continued exploring or remained in a local aggregate. This allowed the swarm to select aggregation behaviors suited to different robot densities. Seeing as swarm robotics enables the simulation of large populations of agents in grounded environments, it is a fitting platform for modeling anthropological theories (Cambier et al., 2020). In particular, it allows ecological parameters such as resource abundance, spatial distribution, and environmental constraints to be systematically manipulated, while measuring their impact on both collective behavior and communication dynamics.

Despite the experimental progress and the potential impact of swarm robotics on language evolution studies, current swarm robotics models lack several crucial features that are considered prerequisites for studying how different socialization patterns shape foraging and communication across environments. Indeed, classic swarm robots are typically highly collaborative and homogeneous, and have little to no memory, not to mention social memory (who did what to whom) (Heinrich et al., 2022). This is in clear contrast with displays from human and non-human primate populations (Roberts and Roberts, 2017) which, by the very nature of our goal, we aim to emulate. Therefore, in order to mimic the potential impact and conditions leading to increased sociality, robots first need to be treated as distinct individuals, and their social tendency needs to be an evolving and flexible parameter that is based on their experience with others. To this end, we designed a novel swarm robotics model of foraging in combination with a language game, which includes two crucial modifications: (1) robot individuation: robots have a partner-specific memory, keeping track of the outcomes of past interactions with specific robots, and (2) parameterizable and evolving sociality: robots' tendency to interact is innate, but fluctuates according to individual experience: successful communication between robots reduces their aggression toward each other and increases their chances of interacting again. This design results in each robot having a unique tendency to interact with each other robot in the swarm, which dynamically changes based on previous interactions.

**Figure 1:**
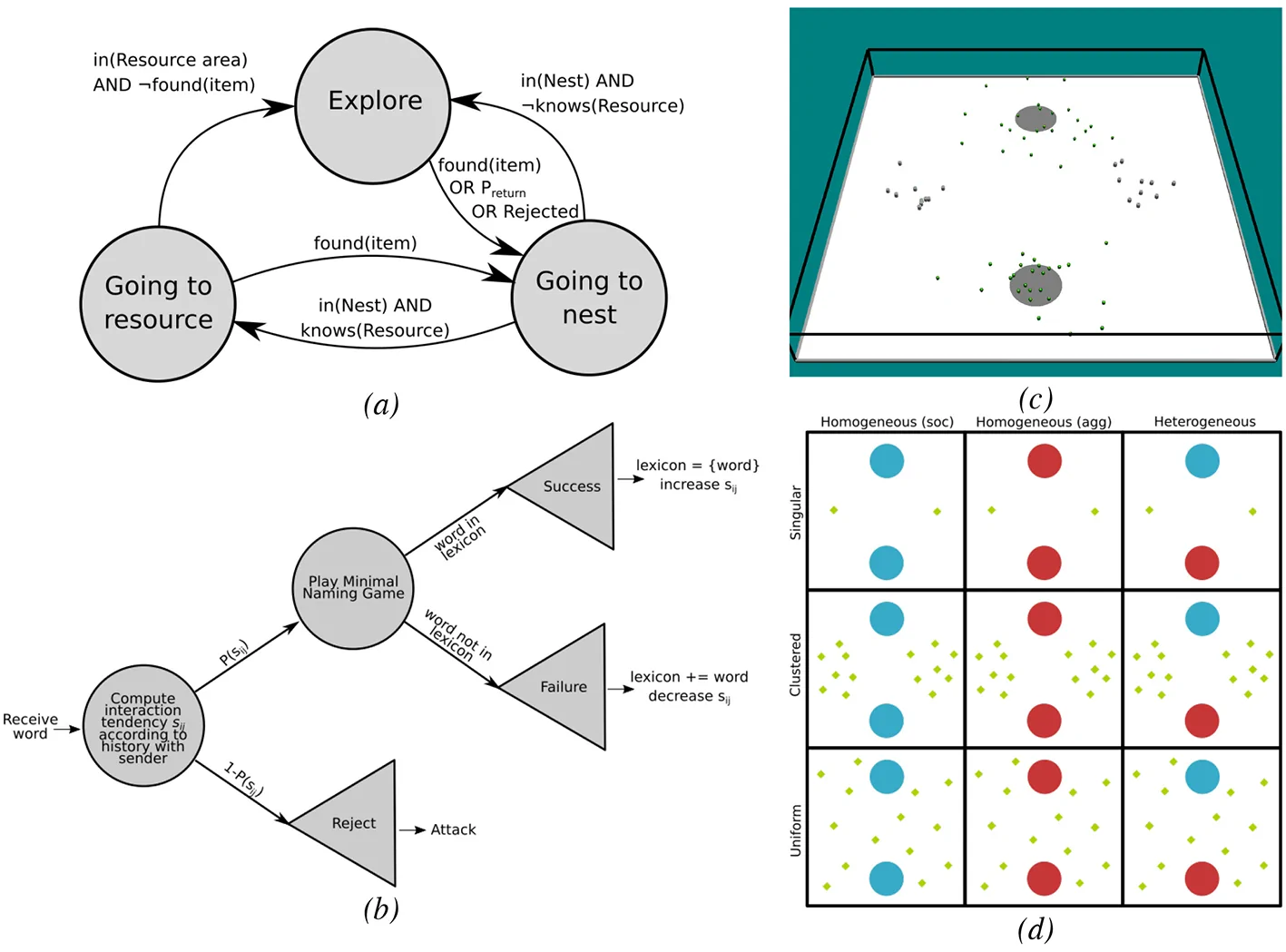
The behavior automaton of the robots guides their foraging strategy **(a)** as well as their interactions **(b)**. Robots explore their environment in search of resources, and when they find an item they take it back to their nest and store the location of the resource as a future target. In any state, a robot can become a *sender* in a Minimal Naming Game (MNG). Another robot in its surroundings can then become a *receiver*, depending on its sociality toward that particular sender. This sociality parameter is based on the receiver's innate tendency (aggressive or social), as well as the outcomes of previous MNGs played with the sender in the past. If the receiver rejects the interaction, it attacks the sender and sends it back to its nest. Robots are embodied in a simulated world **(c)** with realistic physics (e.g., collisions, friction, differential-drive movement, line-of-sight constraints), populated by two nests and randomly distributed food items. We tested robots' social and linguistic behavior in 9 environments **(d)**, with nests inhabited by either aggressive (red) or social (blue) robots, and various distributions (singular, clustered, or uniform) of resource items (green). For each environment, we ran experiments with an *abundant* setup (where a new item spawns as soon as an item is consumed) or a *scarce* setup (where items spawn with a fixed low probability).

As illustrated in Figure 1, our model involves two sub-groups of robots, in a simulated environment with realistic physics, engaged in an underlying foraging task, where they collect resources scattered around their nests. At the beginning of the simulation, each sub-group is geographically situated in one of two different nests. Robots then leave their nest, following random walks, to explore their environment and search for resources, following a probabilistic automaton. During their search for resources, robots also encounter other robots (either from their own nest or from the other nest), and can communicate with each other by playing a Minimal Naming Game (MNG), described in Section 2.2. An important feature of our model is that robots are not able to recognize whether another robot comes from the same nest or not, as we believe this should be an emergent property of our partner-specific memory system. As such, group formation is modular, and depends on robots' own preferences and spatial orientation. We make this choice deliberately, because our goal is precisely to study the emergence of group structure from repeated interaction, rather than to assume it from the outset through explicit group markers or pre-assigned identity tags. We describe our model in more detail in Section 2.

We examine the evolution of the foraging behavior and communicative alignment, within and between nests with two questions in mind: (1) does increased sociality lead to more efficient foraging and thus to better fitness of the nest? And (2) is the effect of increased sociality modulated by the environmental conditions in which robots forage? To this end, we compare, in relevant environments, two “species” of robots with different innate social behaviors: aggressive robots are initially less likely to contribute to the MNG than social robots.

Our social foraging model shows that foraging efficiency and communication dynamics influence each other, and that environments that differ in terms of resource distribution (tightly packed or spread out) and resource availability (scarce or abundant) lead to significantly different foraging and communication behaviors. This parallels hypotheses about human evolution, previously discussed, according to which ecological conditions may have shaped the emergence of social behavior and, in turn, the conditions for communication. Importantly, this article focuses on the transient dynamics of the system rather than on its asymptotic end state. The goal of the present study is therefore to understand how ecological conditions influence this transient phase, and how advantages of sociality may emerge within it. To foreshadow our results, our model suggests that sociality can be beneficial in both scarce and abundant environments, but that its advantages emerge through different dynamics depending on the ecological niche.

## 2 Materials and methods

In our setup, the robots are distributed over two nests (marked on the floor as gray disks) and are set to a foraging task: each robot explores their environment, aiming to retrieve items from resources scattered around their environment and bring them back to their nest.

### 2.1 Foraging

We implemented a probabilistic finite state machine (PFSM), shown in Figure 1a, which guides the robots' behavior. This PFSM is composed of three states:

• Exploring the robot explores its surroundings by following a random walk.
• Going to nest the robot moves toward the position of its nest.
• Going to resource the robot moves toward the position of a known resource.

Notably, this PFSM requires the robot to remember landmark positions and to navigate toward them. This can be achieved through odometry, that is, by estimating a robot's position and orientation from its own movements, typically by integrating wheel rotations over time. Because such estimates can accumulate error, robots may also rely on shared information to improve their position estimate. For example, in social odometry, robots use local information from nearby neighbors such as their position estimates as additional support for localization (Gutiérrez et al., 2009b; Miletitch et al., 2013) on top of their own odometry. In a dense enough swarm, this reduces the accumulation of odometric error over time.

When exploring, robots return to the nest when they have found an item, if they are rejected during an interaction with another robot, or with a probability *P*_*return*_ = 0.0001. This latter scenario ensures that exploration is centered around the nests, and allows experiments in an open environment. Once a robot has reached its nest, it goes back to either exploring, or moving toward a resource if it knows one. If the robot encounters an item on its path toward its memorized resource position, it picks it up, brings it back to the nest and updates the location of its resource in memory. Otherwise, if the robot manages to reach the resource position in memory without encountering any items, it forgets the resource position and switches to the exploring state.

Robots can learn about new resources either by reaching an item (discovery) or by hearing a message from another robot (by playing a language game).

### 2.2 Minimal Naming Game

In parallel to foraging, the robots are playing a Minimal Naming Game (MNG) (Loreto et al., 2010) with local neighbors.

The Minimal Naming Game (Steels, 2011) is a language game in which each robot has its own lexicon, represented here as a set of 8-bit words. Through repeated local interactions, robots in a well-mixed swarm can converge on a shared word. In each game, one robot acts as the sender and another as the receiver. At each time step, each robot has a probability *P*_*send*_ of becoming a sender. A nearby robot may then act as the receiver, depending on its sociality toward that sender, as detailed in Section 2.3.

When a MNG begins, the sender selects a word from its lexicon. If its lexicon is empty, it first creates a new random word. The sender then sends this word to the receiver. If the receiver already has the word in its lexicon, the interaction is successful: both robots delete any other words and retain only that word in their lexicons. Otherwise, the interaction fails: the receiver adds the word to its lexicon, whereas the sender does not modify its lexicon. The receiver then signals the outcome to the sender. This procedure is summarized in Algorithm 1.

Algorithm 1 Minimal Naming Game between sender *i* and receiver *j*.

 Require:  Sender *i*, receiver *j*, with respective lexicon *L*_*i*_, *L*_*j*_ 
       Sender side 
 1:  if *L*_*i*_ = ∅ **then** 
 2:     create a new random word *w* 
 3:     *L*_*i*_←{*w*} 
 4:  else 
 5:     choose a random word *w* ∈ *L*_*i*_ 
 6:  end **if** 
 7:  send *w* from *i* to *j* 
  Receiver side 
 8:  if *w* ∈ *L*_*j*_ **then** 
 9:     *L*_*j*_←{*w*} 
 10:     signal success to *i* 
 11:  else 
 12:     *L*_*j*_←*L*_*j*_∪{*w*} 
 13:     signal failure to *i* 
 14:  end **if**

### 2.3 Partner-specific sociality

The novelty of our approach is that we introduce a sociality factor, which dictates the communications between robots based on social memory. As a result, sub-groups may converge fully, or alternatively stop communicating almost entirely and maintain separate languages, as can be observed in human populations (Bickel and Nichols, 2009). This social memory is achieved by the introduction of two novel socio-cognitive features, previously absent from classic swarm robotics models inspired by social insects. The first feature is robot individuation, whereby robots have a partner-specific memory and keep track of the outcomes of past interactions with other robots. Crucially, robots are not able to tell whether or not another robot comes from the same nest as them. Instead, their affinity with specific robots is based on their previous experience. Let $mng_{ij}^{k}\in \left\{0,1\right\}$ denote the outcome of the *k*-th MNG between robots *i* and *j* (1 for a success, 0 for a failure). We define *K*_*ij*_ as the total number of MNGs played between robots *i* and *j*, and let robot *i* maintain a fixed-size memory window $B_{ij}^{K_{ij}}$ of the last *w* MNGs played with robot *j*:


$$\begin{array}{l} B_{ij}^{K_{ij}}=(mng_{ij}^{K_{ij}},mng_{ij}^{K_{ij}-1},\ldots ,mng_{ij}^{K_{ij}-w+1}) \end{array}$$ (1)

The second feature is flexible (or evolving) sociality: robots' tendency to interact with others (i.e., their willingness to accept the role of receiver in an incoming MNG) is not fixed, but changes over time. At the beginning of the experiment, robots from a given nest are assigned a predetermined (i.e., “innate”) sociality value, which can be relatively high or relatively low, corresponding to social or aggressive robots. However, as robots move through the environment and start interacting with others, this value will change based on experience with other robots, such that robots have a partner-specific sociality value. Successful communication between two robots increases their sociality toward each other (and therefore their willingness to communicate again), while a failed communication decreases their sociality toward each other. Additionally, after a failed interaction, the robot that started the interaction and got rejected is forced to return to its nest (see Figure 1a). This rule models an aggressive rejection: after being refused, the sender is driven away from the local area and retreats toward its nest, as may occur following a competitive encounter.

More specifically, we compute sociality (i.e., the probability that robot *i* accepts an interaction initiated by robot *j*) *s*_*ij*_(*K*_*ij*_, *t*) as a weighted moving average of the number of successful past interactions between robots *i* and *j*:


$$\begin{array}{l} s_{ij}\left(K_{ij},t\right)=\frac{1}{w}\overset{K_{ij}}{\underset{k=K_{ij}-w+1}{\sum }}\left(d_{ij}^{k}\times mng_{ij}^{k}+\left(1-d_{ij}^{k}\right)\times s_{i}^{0}\right),\\ \;\text{ with }d_{ij}^{k}=e^{-\text{λ}\left(t-t_{ij}^{k}\right)},\;mng_{ij}^{k}\in \left\{0,1\right\} \end{array}$$ (2)

where $s_{i}^{0}$ is the baseline sociality of robot *i*, $t_{ij}^{k}$ the time of the *k*-th interaction between both robots, *w* the memory window size (in our model, *w* = 10), and λ the memory decay parameter (in our model, λ = 0.00001). The latter controls how quickly the influence of past interactions fades, gradually pulling *s*_*ij*_(*K*_*ij*_, *t*) back toward the baseline value $s_{i}^{0}$. Crucially, λ does not behave like a probability, but is part of a weight that can increase or decrease the impact of memorized interactions over time. Overall, the memory window size *w* determines how many recent interaction outcomes contribute to the sociality estimate: small values make sociality more responsive to individual encounters, whereas large values smooth changes across more experiences. The decay parameter λ determines how quickly past outcomes lose influence: high values return sociality toward its baseline more rapidly, whereas low values preserve the effects of past interactions for longer. Together, these parameters control the speed and persistence of social dynamics; a more detailed analysis is provided in Miletitch and Raviv (2026). To stabilize early dynamics, we introduce a prior consisting of a pre-filled backlog of interactions. We make them infinitely old, hence neutral toward the baseline sociality $s_{i}^{0}$ because of decay. It is worth noting that, once linguistic convergence is reached across the whole swarm, subsequent MNGs can no longer reduce sociality through failure. Sociality therefore can only increase and robots (whether initially social or aggressive) gradually become a single cohesive swarm.

Finally, robot individuation creates the need for the aggregation of individual values to obtain accurate statistics at the swarm level. We can aggregate robots based on their initial innate sociality (aggressive or social) and their relationships (whether they interact with a robot within their sub-group or outside it). This results in the following standard statistical formula for averaging Interactions, MNG games played, and sociality, and is applicable to either robots of the same nest (in-group, *X*^*in*^) or from the other nest (out-group, *X*^*out*^), for either aggressive or social agents:


$$\begin{array}{cc} X_{agg}^{in}=\frac{\sum_{i\in I_{agg}}\sum_{j\in I_{in}\wedge j\neq i}x_{ij}}{\left|I_{agg}|+|I_{agg}\right|}, & \;X_{soc}^{in}=\frac{\sum_{i\in I_{soc}}\sum_{j\in I_{in}\wedge j\neq i}x_{ij}}{\left|I_{soc}|+|I_{soc}\right|}\\ X_{agg}^{out}=\frac{\sum_{i\in I_{agg}}\sum_{j\in I_{out}}x_{ij}}{\left|I_{agg}|+|I_{soc}\right|}, & \;X_{soc}^{out}=\frac{\sum_{i\in I_{out}}\sum_{j\in I_{agg}}x_{ij}}{\left|I_{soc}|+|I_{agg}\right|}\\ \text{where }X\in \left\{Inter,MNG,S\right\} & \text{and }x\in \left\{inter,mng,s\right\} \end{array}$$

with x being the value on the robot level and X the resulting statistic on the swarm level, *I*_*agg*_ and *I*_*soc*_ representing the lists of robot indices respectively from the aggressive swarm and the social swarm, and *I*_*in*_ and *I*_*out*_ the lists of robot indices respectively from their in-group and their out-group. *Inter*, *MNG*, and *S* refer respectively to the number of interactions, the number of MNGs played, and the sociality value.

### 2.4 Experimental setup

In order to test our hypotheses, we set up a swarm of 50 robots (25 per nest) in an enclosed arena of 10 m × 10 m with two nests in the center and two resources around, as spread of items. Specifically, the arena size was designed to be large enough that robots guided by the behavior detailed in Figure 1a would almost never reach its limits,^1^ so it is assimilable to an open environment. Both nests are 1.2 meters in diameter, and 6 meters apart from one another. Resources are equally distant from each nest, and 6 meters apart from one another. The item distribution of each resource follows a Gaussian curve centered on the resource, with a standard deviation σ ∈ {0.01, 1, 10}, respectively named *singular, clustered*, and *uniform*. Each resource can contain a maximum of 25 items. Whenever a resource is not full, items will spawn with a probability *p*_*item*_ ∈ {1, 0.005}, respectively named *abundant* and *scarce* conditions. Robots start with an initial sociality value *s* ∈ {0.1, 0.9}. For consistency with previous research (Cambier et al., 2022), we set λ = 0.00001 for the memory decay parameter and *w* = 10 for the memory window size, as these parameter values yield dynamics similar to those observed in earlier studies. This makes comparisons more straightforward and allows the present work to be interpreted as an extension of that framework.

For each experimental setup, we ran 100 runs, carried out with ARGoS3 (Pinciroli et al., 2012), a physically realistic simulator made for swarm robotics experimentation, with a simulation rate of 10 ticks per second. We simulated e-puck robots [more information about the robot can be retrieved in Mondada et al. (2009) and Garattoni et al. (2016)], a robot that uses a range-and-bearing (RAB) system to communicate locally (Gutiérrez et al., 2009a). All parameters are summarized in Table 1.

**Table 1:** Experimental parameters.

General setup	Value(s)	Description
Arena size	10*m*×10*m*	Size of the square arena
Nests	2	Number of nests
Nest population	25	Number of robots per nest
Nest distance	6*m*	Distance between nests
Nest size	1.2*m*	Diameter of nest area
Environment	Value(s)	Description
Source Distance	6*m*	Distance between resource loci
Items	25	Max items per resource
Renewal		Item spawning probability
Abundant	1*s*^−1^	
Scarce	0.005*s*^−1^	
Spread		Standard deviation of items distribution
Singular	0.01*m*	
Clustered	1*m*	
Uniform	10*m*	
Behavior	Value(s)	Description
*speed*	10*cm*/*s*	Maximum agent speed
*P*_*return*_	0.0001	Probability to return to the nest
*P*_*send*_	0.01	Probability to speak
$s_{agg}^{0}$	0.1	Initial sociality for aggressive agents
$s_{soc}^{0}$	0.9	Initial sociality for social agents
λ	0.00001	Memory decay parameter
*w*	10	Memory window size
Robot	Value(s)	Description
Robots	e-puck	Robot model
Diameter	70*mm*	Robot body diameter
Height	45*mm*	
Weight	130*g*	
Wheel	41*mm*	Wheel diameter
Inter-wheel	53*mm*	
RAB range	0.5*m*	
Control rate	10*Hz*	
Resource detection radius	30*mm*	

Each run lasts 10,000 simulated seconds. While this may seem short compared to naturalistic observations that can extend over days or weeks, our aim here is not to reproduce the full long-term trajectory of a population, but rather to isolate and compare the transient dynamics of the model across conditions. This choice also matches the practical constraints of simulation experiments, and keeps the setup closer to future implementations with real robots, where shorter and controlled trials are more realistic.

## 3 Results

For each scenario, we report three outcomes: (1) sociality (*S*), calculated separately for robots from the same nest (in-group) and from the other nest (out-group); (2) interaction and MNG rates (*Inter* and *MNG*); and (3) item-collection rate per nest. The experimental procedure is described in Section 2; in particular, the definitions of *S*, *Inter*, and *MNG* are provided in Section 2.3.

We first describe the main foraging and linguistic dynamics. We then examine the homogeneous scenarios in detail, first analyzing the evolution over time of interactions, MNGs, and sociality across social and environmental conditions, and then comparing item-collection rates. Finally, we turn to the heterogeneous scenario, beginning with an overview of interaction, MNG, and sociality dynamics before focusing on item-collection performance. We then finish with a discussion of our results.

### 3.1 Overview

Two major factors shape these results. Firstly, the distribution of the two sub-groups in space, and in particular their spatial proximity, is impacted by how the resources are spread. Secondly, the convergence on vocabulary, and hence how likely it is for the robots' interactions to result in successful communications.

#### 3.1.1 Item distribution

Differences in the spread of resources yield different swarm topologies, and lead to different interaction patterns and item-collection rate. Specifically, clustered and singular resources can promote spatial separation between sub-swarms, as rejected out-group robots return toward their own nest, making it more likely that the two groups exploit different resource patches. Robots are therefore less likely to cross paths and, hence, to interact. At the same time, in the singular distribution, robots converge to well-defined areas resulting in denser local interaction networks. Meanwhile, the uniform distribution reduces interactions overall as robots can fetch items anywhere in the environment.

Comparatively, social robots are not deterred and explore the environment more widely. This exploration is especially advantageous when resources are abundant, and robots are not limited in their exploitation of resources. On the other hand, with the uniform distribution, robots spread across the arena and do not interact as much, which reduces differences in collection performance between the social and aggressive types. This happens especially in the long term, as at the beginning of the simulation, aggressive robots are rejected more frequently and therefore return to their nests more often, keeping them close to the nests for longer.

In environments with clustered or uniform distributions that are resource-scarce, this dynamic is not significant since resources renew more slowly than they are exploited. However, the limitation from aggressive behavior is notably evident in singular resource scenarios and remains relevant here, even as resources are scarce.

**Figure 2:**
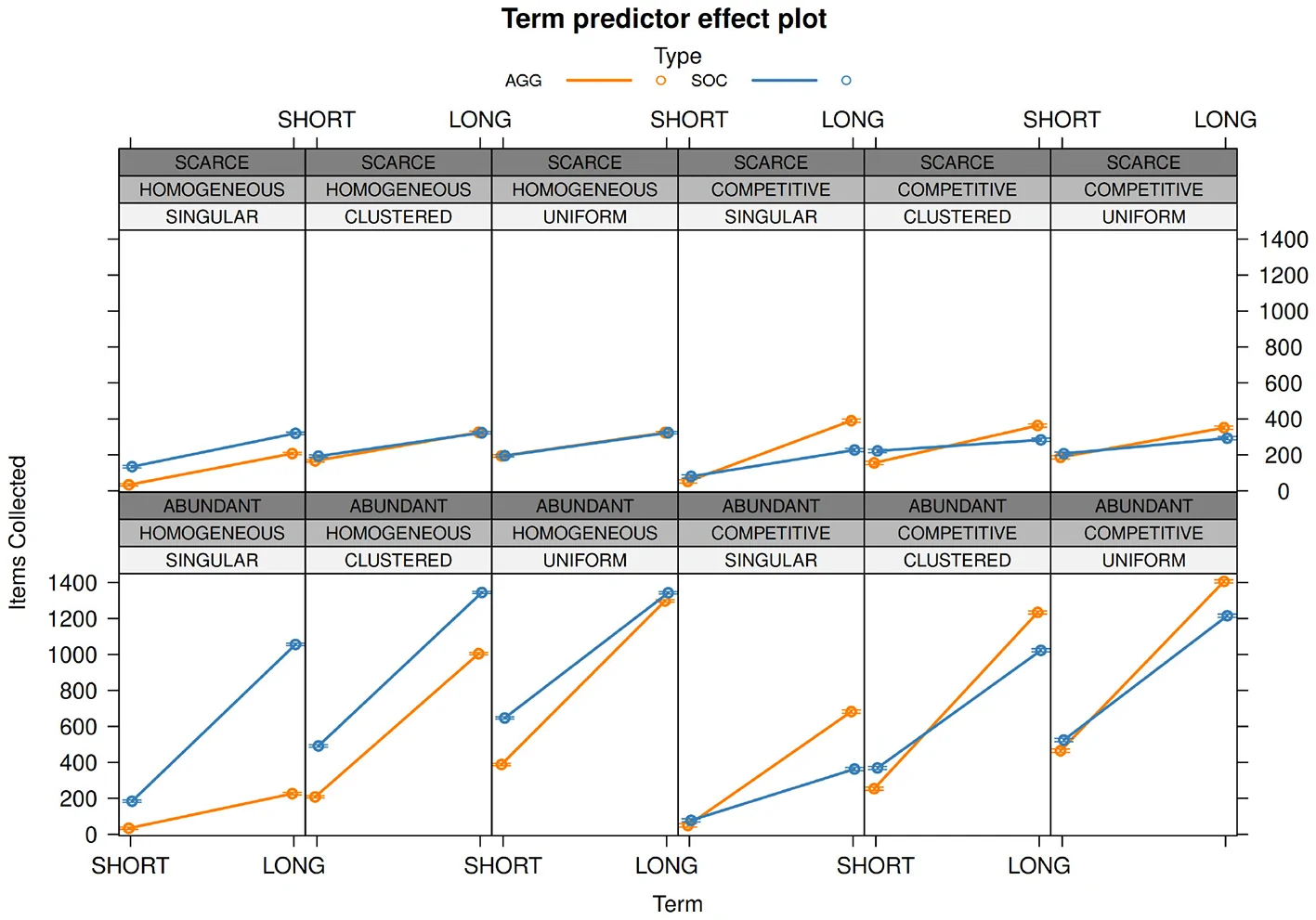
Fitted values of cumulative item collection from the linear mixed-effects model for aggressive (AGG) and social (SOC) robots across short- and long-term periods. Panels correspond to combinations of resource availability (scarce or abundant), scenario composition (homogeneous or heterogeneous), and resource distribution (singular, clustered, or uniform). Points represent the fitted means estimated by the model, lines connect short- and long-term values, and error bars indicate 95% confidence intervals. Short and long terms respectively refer to the periods before and after aggressive agents, with an initial sociality of 0.1, reach an in-group sociality of 0.9 in the corresponding experimental condition.

To quantify these patterns, we fitted a linear mixed-effects model in *R* (Figure 2) with cumulative item collection in each nest as the dependent variable. Fixed effects included robot type (*aggressive* vs. *social*), resource spread (*singular, clustered, uniform*), term (*short* vs. *long*), scenario composition (*homogeneous* vs. *heterogeneous*), and resource availability (*abundant* vs. *scarce*), together with their interactions. A random intercept for simulation seed was included to account for repeated observations derived from the same run. This model tests whether differences in foraging performance between aggressive and social robots remain constant across environments, or instead depend on resource structure and social context.

For the “term” variable, two time-frames were considered: short- and long-term. Short-term corresponds to the time-frame between the beginning of the experiments and the time-step where aggressive agents reach an in-group sociality of 0.9 (i.e., they become as social with their in-group as social agents were at the beginning of the experiment). Long-term is the time-frame between this time-step and the end of the experiment. For heterogeneous scenarios, where one nest is inhabited by aggressive agents and the other is inhabited by social agents, this cut-off point is unique to each experimental run. It corresponds to the moment where the agents within the aggressive nest reach an average sociality of 0.9 with other in-group agents. However, for half of the homogeneous scenarios, where both nests are made up of social agents, this cut-off point is impossible to determine. In those cases the cut-off point was determined as the cut-off point in the equivalent scenario with aggressive agents, and the same random seed, which guarantees the same experimental conditions regarding the initial placement of both agents and items.

The complete results of the linear mixed-effects model, including the estimated coefficients, standard errors, and (**t**)-values for all main effects and interaction terms, are reported in Supplementary Table S1.

#### 3.1.2 Vocabulary convergence

Before the convergence of a sub-group on a common vocabulary, any MNG being played has the potential to lower the sociality between robots through failure. As convergence on a common vocabulary progresses, a growing proportion of MNGs are successful, which in turn increases sociality, until communication within the sub-group becomes almost exclusively successful. The time to lexical convergence therefore closely tracks the transient social phase of the system and helps determine its duration.

Importantly, the naming game is not an accessory component of the model, but is rather the mechanism through which communication outcomes reshape partner-specific sociality over time. This influence runs in both directions: the outcomes of the naming game reshape sociality, and sociality in turn affects how quickly and how locally vocabularies converge. A setup with fixed sociality values and explicit group membership could still reveal differences in foraging under different behavioral regimes, but it would not capture the emergence of group structure from interaction, nor the feedback loop by which foraging opportunities, communicative alignment, and social behavior jointly organize one another.

In the following, we first examine the evolution of sociality, then its consequences for interaction and MNG rates, and finally the resulting linguistic dynamics, as each level constrains the next.

**Figure 3:**
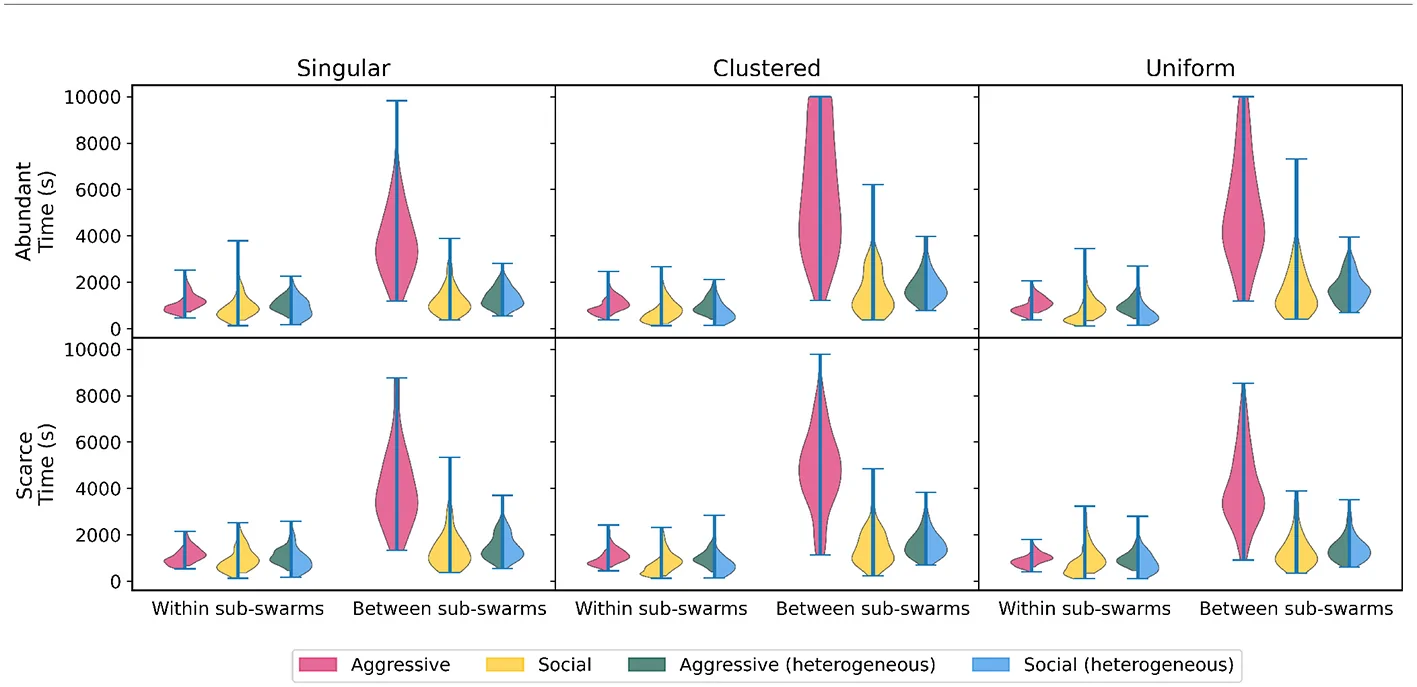
Time to converge on a single word, within and between sub-groups, per sociality and different initial sociality (whether both sub-groups have the same initial sociality or not). Violin widths represent the distribution of convergence times across simulations. Colors distinguish initially aggressive and social robots in homogeneous and heterogeneous swarms. Bars indicate 95% confidence intervals. In homogeneous cases, the convergence within sub-groups aggregates the times for the first nest to converge (in each run) on the left and the last one on the right. Overall, sub-swarms converge internally before reaching convergence across the whole swarm. In heterogeneous scenarios, social robots also tend to converge faster than aggressive robots.

Figure 3 plots the convergence times, per nest, for all scenarios (environment and sociality). Within nests, the convergence time is clearly bimodal, meaning that the two nests do not converge simultaneously. This is surprising in the homogeneous case as the environment is mostly symmetrical (resource locations are at equal distances from the nest, although item distribution may favor one side). We explain this distribution by the fact that, once a nest has converged on a shared lexicon, it consistently injects its word in the lexicon of the other nest, thus delaying its convergence. This explanation is reinforced by looking at the convergence times between nests, which show that one nest tends to converge faster and then to propagate its word to the other nest, as the convergence of the second nest and the between-nest convergence have similar distributions. Aggressive agents tend to converge locally before doing so on the global level (on a much larger time scale). Notably, this inter-swarm lexical convergence seems to be even more delayed when resources are not singular, as it leads to more interaction between robots.

Finally, heterogeneous scenarios are often quite close to social homogeneous scenarios, since social agents have more influence over the sociality of aggressive agents than the opposite. However, the in-group convergence is generally as fast as with purely aggressive agents, as aggressive agents induce a strong separation of the population before convergence.

### 3.2 Homogeneous scenario

**Figure 4:**
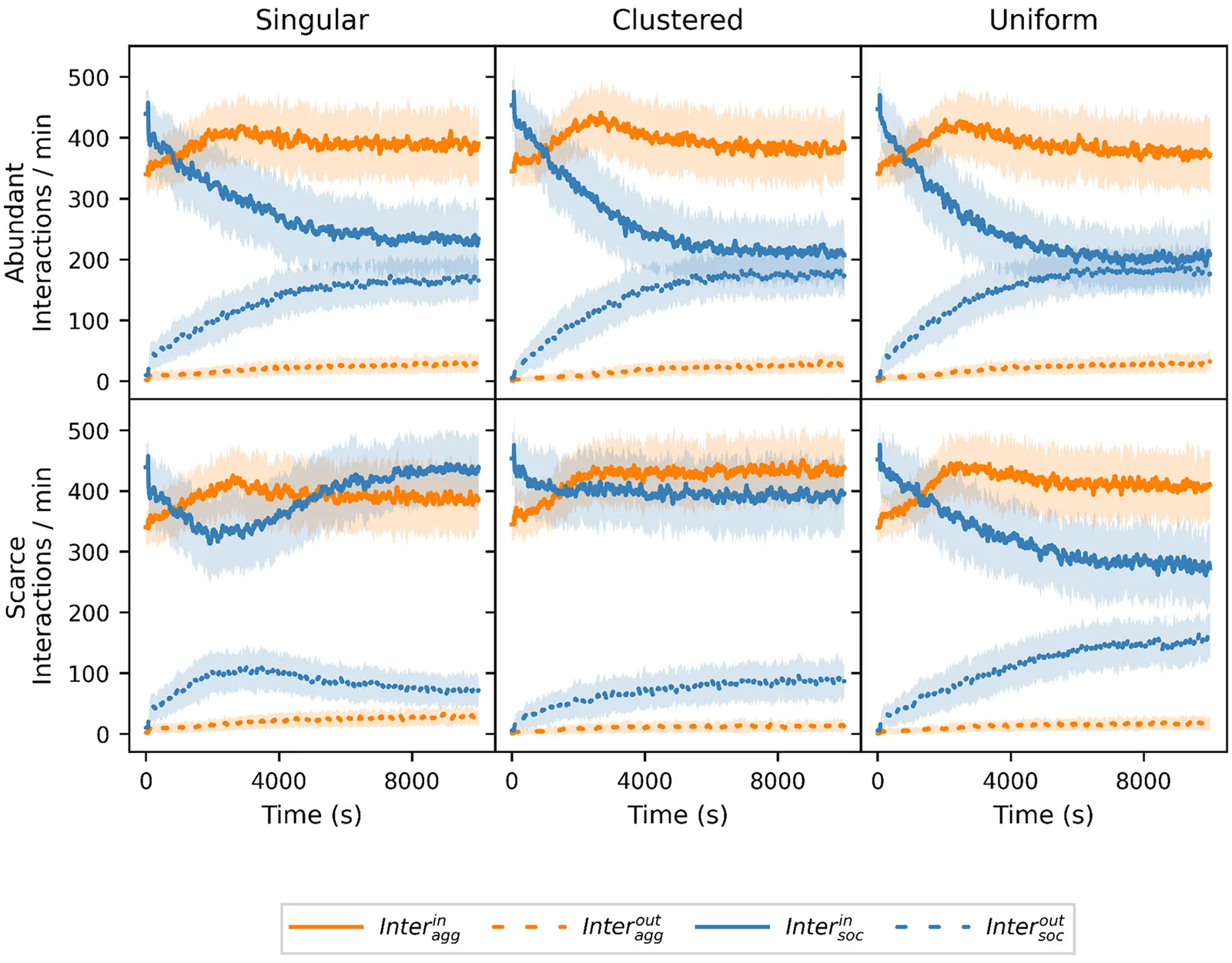
Evolution of the total rate of interactions between agents over time for different item distributions (columns), item availability (rows) and initial sociality (colors). Both sub-groups have the same initial sociality. Lines show mean values across 100 simulation runs, and shaded areas indicate ±1 standard deviation. Subscripts *agg* and *soc* refer to aggressive and social robots, respectively, whereas superscripts *in* and *out* refer to interactions with in-group robots and out-group robots, respectively.

Figure 4 shows the number of interactions between agents. As they spawn at the beginning of the simulation in their respective nests, each sub-group starts out with a high number of in-group interactions and a low number of out-group interactions. That is, the range of communication of the robots is quite restrained, implying that robots only communicate with the robots around them, i.e., from their in-group. Since, by design, aggressive agents trigger rejections (i.e., failed interaction) more regularly than social agents and thus return to their nest more often, compared to the latter—they remain around their nest, and have fewer chances to interact with robots from outside their sub-group. Over time, this spatial bias toward in-group interactions weakens as robots can explore farther from their nests and encounter out-group robots more frequently.

This in-group interaction bias becomes more pronounced as resources are more spatially spread. An exception appears in the abundant, singular resource condition. In this case, once the simulation has progressed enough, most social robots have converged around the highly localized resources, increasing local swarm density and, consequently, the number of interactions between robots anew.

**Figure 5:**
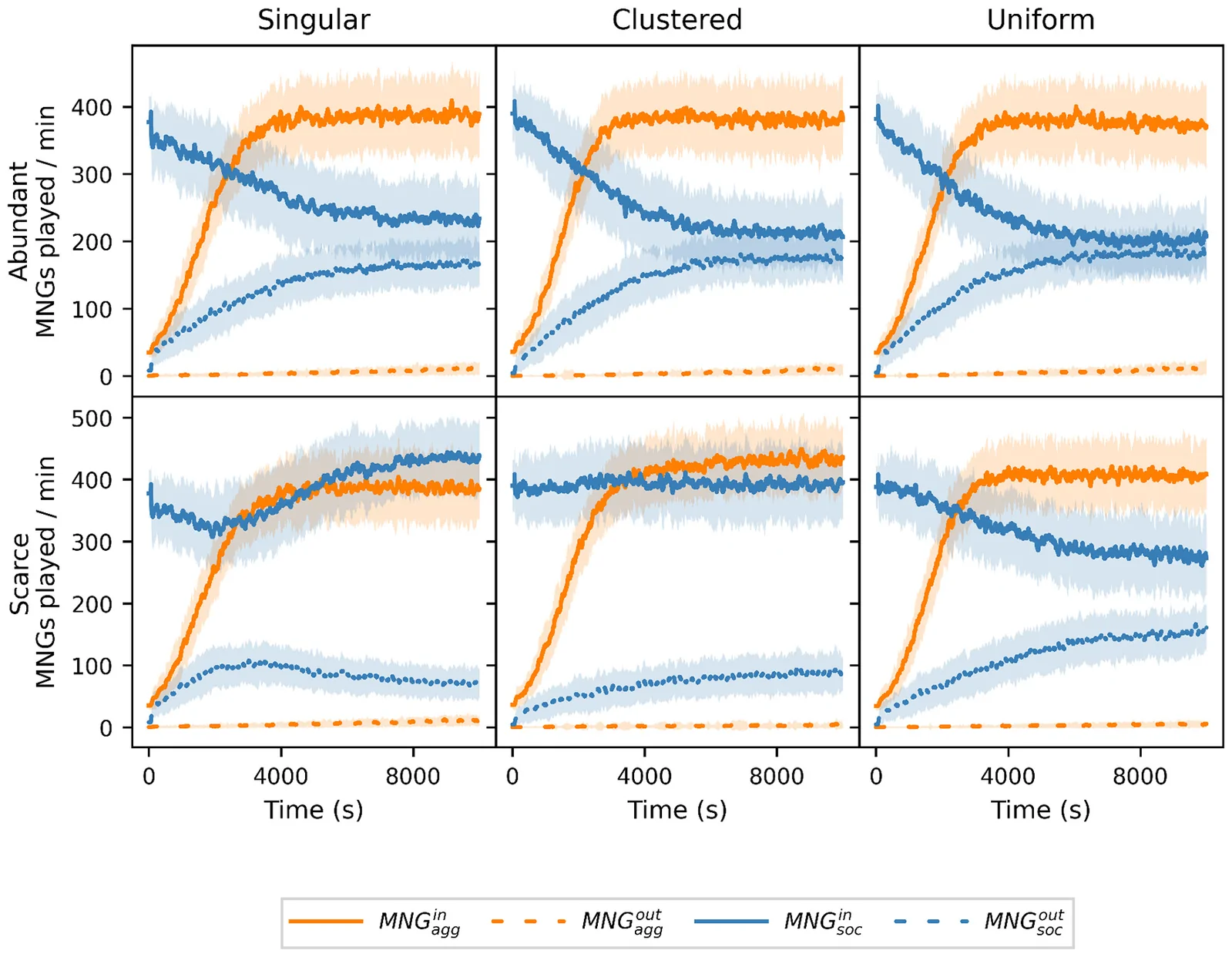
Evolution of the total rate of naming games played over time for different item distributions (columns), item availability (rows) and initial sociality (colors). Both sub-groups have the same initial sociality. Lines show mean values across 100 simulation runs, and shaded areas indicate (±1) standard deviation. Subscripts *agg* and *soc* refer to aggressive and social robots, respectively, whereas superscripts *in* and *out* refer to interactions with in-group robots and out-group robots, respectively.

Figure 5 shows the evolution of the rate of MNGs played. A high sociality implies that every interaction ends up with an MNG being played. This is why the curve associated with social robots is virtually the same here as in Figure 4. This is particularly explicit in the case of a scarce environment, where robots have fewer interactions. Aggressive robots, on the other hand, have a transitory period where they have not yet converged upon a common vocabulary, and hence not all interactions conclude in a played MNG.

For social robots, the extent to which the initial in-group interaction bias weakens depends on the environmental conditions. It decreases most clearly under scarce resources, where depletion promotes continued exploration, and also in the abundant-uniform condition, where items are widely dispersed. In contrast, abundant singular and clustered resources allow repeated exploitation of localized areas, helping maintain higher in-group interaction rates. In the abundant-singular condition, the temporary decrease in in-group interactions (dip in the solid blue line in the abundant-singular panel of Figure 4) is consistent with robots initially spreading between the two resource locations before later concentrating on one.

**Figure 6:**
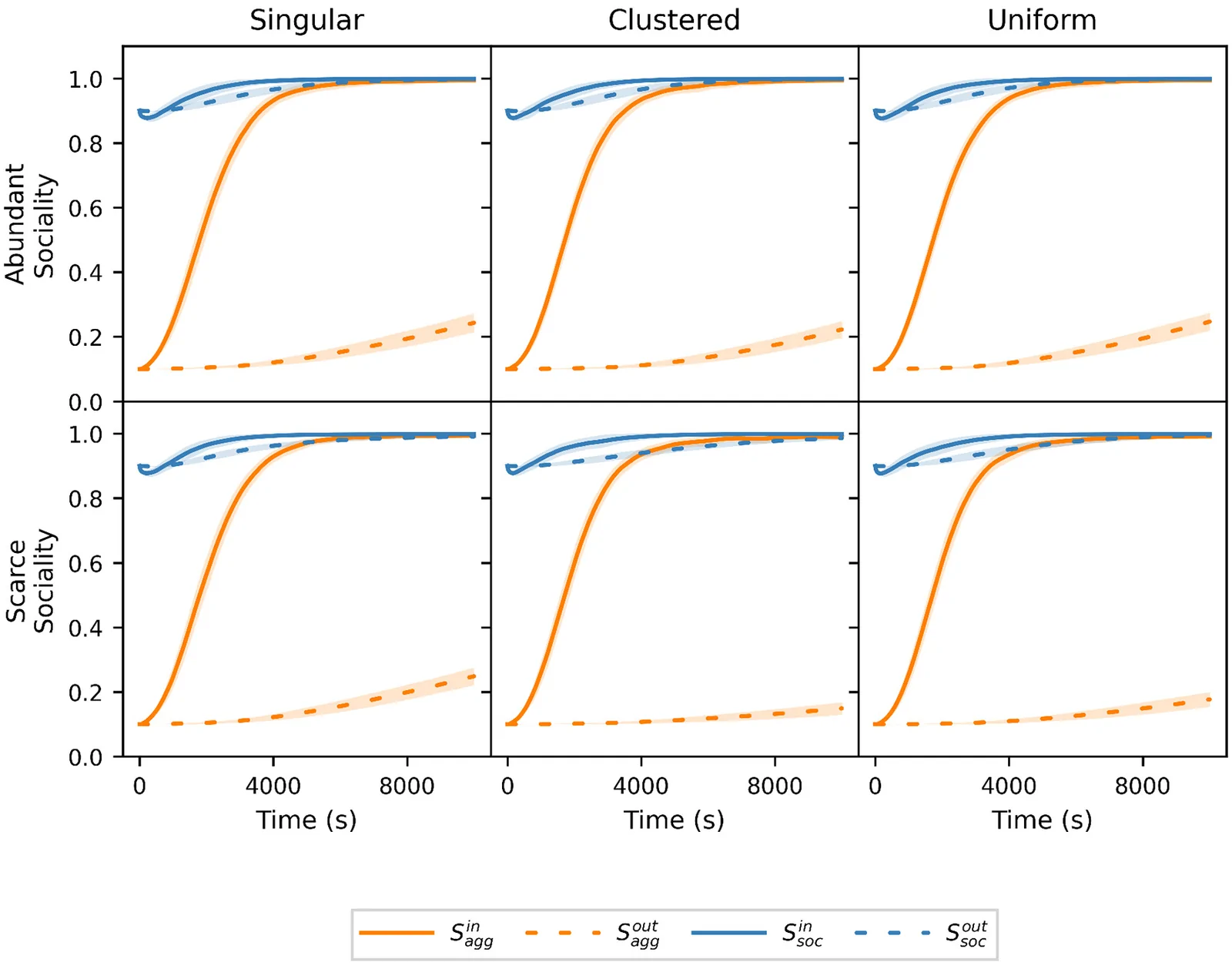
Evolution of the sociality over time for different item distributions (columns), item availability (rows) and initial sociality (colors). Both sub-groups have the same initial sociality. Lines show mean values across 100 simulation runs, and shaded areas indicate (±1) standard deviation. Subscripts *agg* and *soc* refer to aggressive and social robots, respectively, whereas superscripts *in* and *out* refer to interactions with in-group robots and out-group robots, respectively.

When both sub-groups start out with low initial sociality, most initial interactions would be refused regardless of the potential outcome of the MNG. Thus, when both sub-groups start out as aggressive, no MNGs are being played at the start of the experiment. Yet as robots' in-group sociality increases over time (see Figure 6), more of these interactions end up in a MNG being played, and eventually reaches a plateau once in-group sociality is at ceiling and robots have converged on a shared vocabulary (as seen in Figure 3). Notably, as per Figures 4, 5, aggressive robots seem to settle on having nearly exclusively in-group communication, while social agents interact more evenly within and across sub-groups.

The evolution of the sociality is shown in Figure 6. The small initial dip in the solid blue curve in each panel reflects a temporary decrease in social robots' in-group sociality. This occurs because, before converging on a shared language (Figure 3), even social robots experience frequent unsuccessful MNGs within their own group. Soon, they become more open to their in-group and, eventually, to the out-swarm. In contrast, aggressive agents develop a very clear in-group preference (i.e., high sociality toward the in-group, but low sociality toward the out-swarm) due to having few interactions outside of their nest. This in-group bias is slightly less pronounced (orange dotted line increasing more rapidly) in environments wherein items are more difficult to find (singular and/or scarce distributions), as robots find fewer items and therefore explore more and encounter more out-swarm robots (see Figure 4).

It is important to note that the behavior of the aggressive swarm is asymptotic (as is the one of the social swarm) and, as such, would rejoin the social swarm in a convergence of *s* = 1. Since our current model focuses on a positive social feedback loop to study how social behavior can impact communication processes, such convergence, if slow, is to be expected. This article focuses solely on the transitory dynamics and how the speed of each (language on one hand, and social behavior on the other) impacts the overall behavior.

**Figure 7:**
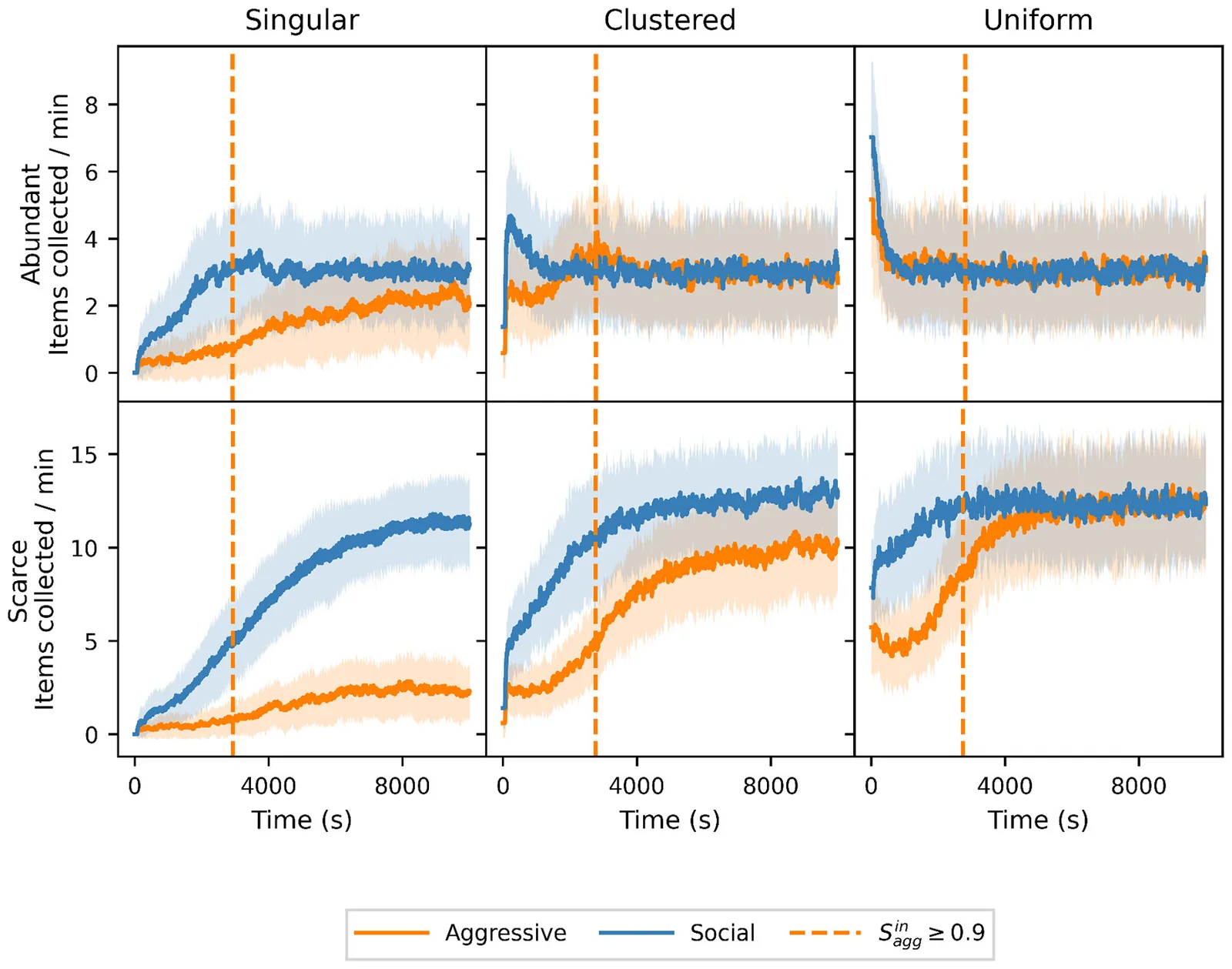
Evolution of the rate of collected resource items over time (average per nest) for different item distributions (columns), item availability (rows) and initial sociality (colors). Both sub-groups have the same initial sociality. The dashed orange lines indicate the time at which aggressive agents (initial sociality = 0.1) reach an in-group sociality of 0.9. Lines show mean values across 100 simulation runs, and shaded areas indicate (±1) standard deviation.

Finally, Figure 7 shows the average rate of item collection per nest. On average, the rate of item collection is higher for social robots than for aggressive robots, which was already observed in the homogeneous panels of Figure 2. This is especially the case with abundant resources when item spawning probability is high enough to accommodate high rates of collection. This difference in item collection from sociality is lowered over time, as the sociality of aggressive robots rises, and gets closer to that of social robots. Indeed, Figure 7 shows that the difference between social and aggressive robots is largest before the dashed line, and becomes smaller afterwards, when aggressive robots reach an in-group sociality of 0.9. This effect is particularly visible in clustered and uniform item distribution, as they lead to different interaction patterns. In environments with clustered or uniform distributions that are resource-scarce, this dynamic is not significant since resources renew more slowly than they are exploited. However, the limitation from aggressive behavior is notably evident in singular resource scenarios and remains relevant here, even as resources are scarce (as is also seen in the singular resources panels of Figure 2).

### 3.3 Heterogeneous scenario

In this section, we study heterogeneity by introducing two species in the same environment. Specifically, the robots in the sub-groups of each nest have different initial sociality. Therefore, the first nest is inhabited by a sub-group of social robots, while the second nest is inhabited by a sub-group of aggressive robots. This allows us to not only compare the dynamics of each species, but also see how they interact with each other and how they behave in a more complex environment.

**Figure 8:**
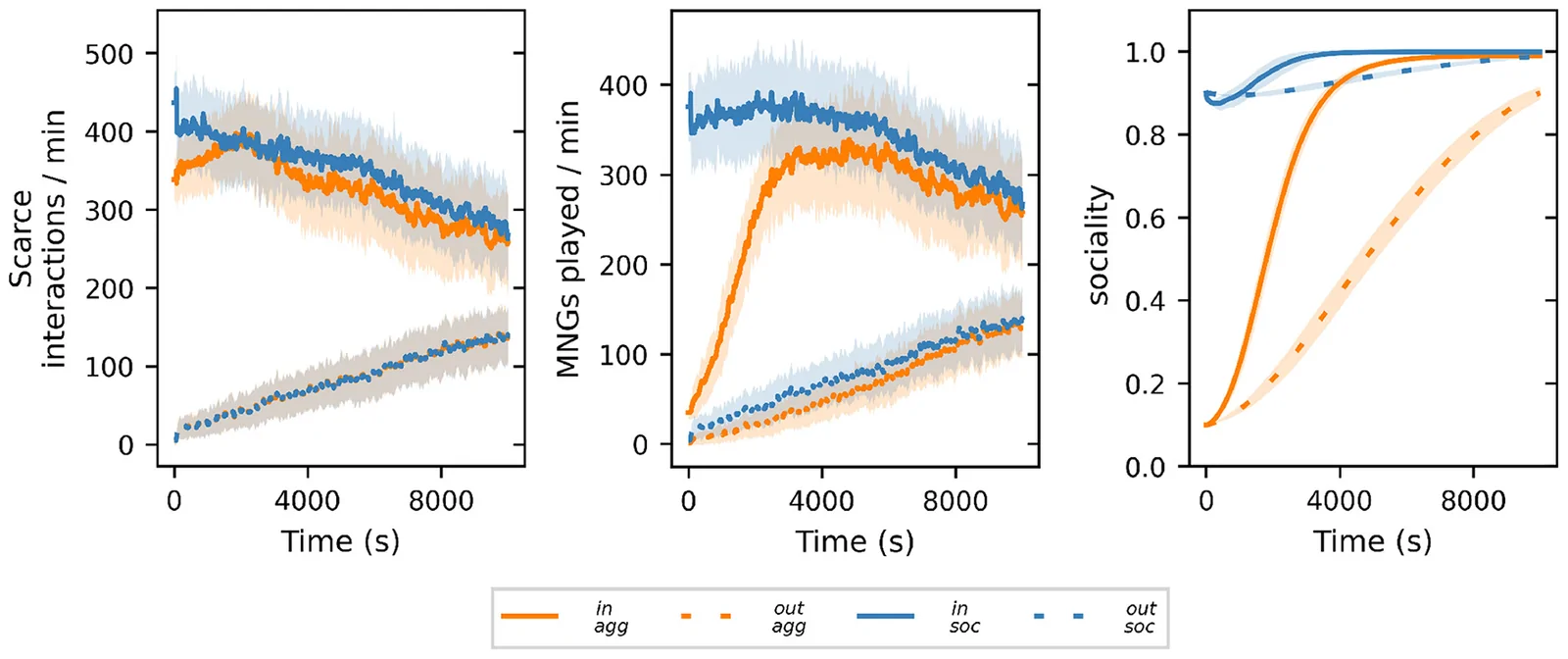
Evolution, per nest, of (from left to right) the rate of interactions played over time, the rate of naming games played over time, and the sociality in the case of a singular scarce resource. The sub-groups have different initial sociality. Lines show mean values across 100 simulation runs, and shaded areas indicate (±1) standard deviation. Subscripts *agg* and *soc* refer to aggressive and social robots, respectively, whereas superscripts *in* and *out* refer to interactions with in-group robots and out-group robots, respectively.

As aggressive robots engage in fewer interactions than social robots, the evolution of the overall interaction rate is driven mainly by the social robots. This is reflected in the interaction-rate panel of Figure 8, which shows a pattern strikingly similar to that observed in the homogeneous setup where both nests initially contain social robots (Figure 4).

The evolution of the number of MNGs being played (middle of Figure 8) behaves similarly, with an evolution over time on par with the social homogeneous case, and declining tendencies because of interaction with aggressive robots, as above.

Although Figure 8 presents the singular-scarce condition as a representative example, the same overall qualitative pattern was observed in both abundant and scarce environments and across all three resource distributions (Supplementary Figures S1–S3). In particular, social robots remained highly social, whereas aggressive robots first increased their in-group sociality and subsequently showed a slower increase in out-group sociality; resource availability primarily modulated the magnitude and temporal evolution of interactions and naming games rather than changing this general pattern.

Similarly, the evolution of in- and out-group sociality as a function of the sub-groups' initial sociality suggests a pattern of results very similar to that seen in the homogeneous social setup. Indeed, the social evolution panel in Figure 8 of social robots mirrors the one seen in the singular scarce resource panel in Figure 6 (social robots remain social, aggressive robots become more social toward their in-group first, then slowly over time to their out-group). One exception is that out-group sociality of aggressive robots rises much faster (orange dotted line of the sociality panel of Figure 8). This can be explained by the fact that in the heterogeneous scenario, the out-group of aggressive robots is, in fact, composed of social robots—who are more eager to engage with the aggressive robots. For the same reason, the growth of the social robots' out-group sociality is slightly slower than in the homogeneous scenario, seeing as their out-group is comprised of more aggressive robots. Notably, the convergence dynamic of the MNG was largely unaffected by the kind of scenario we are running (would it be environmental conditions or sociality level).

**Figure 9:**
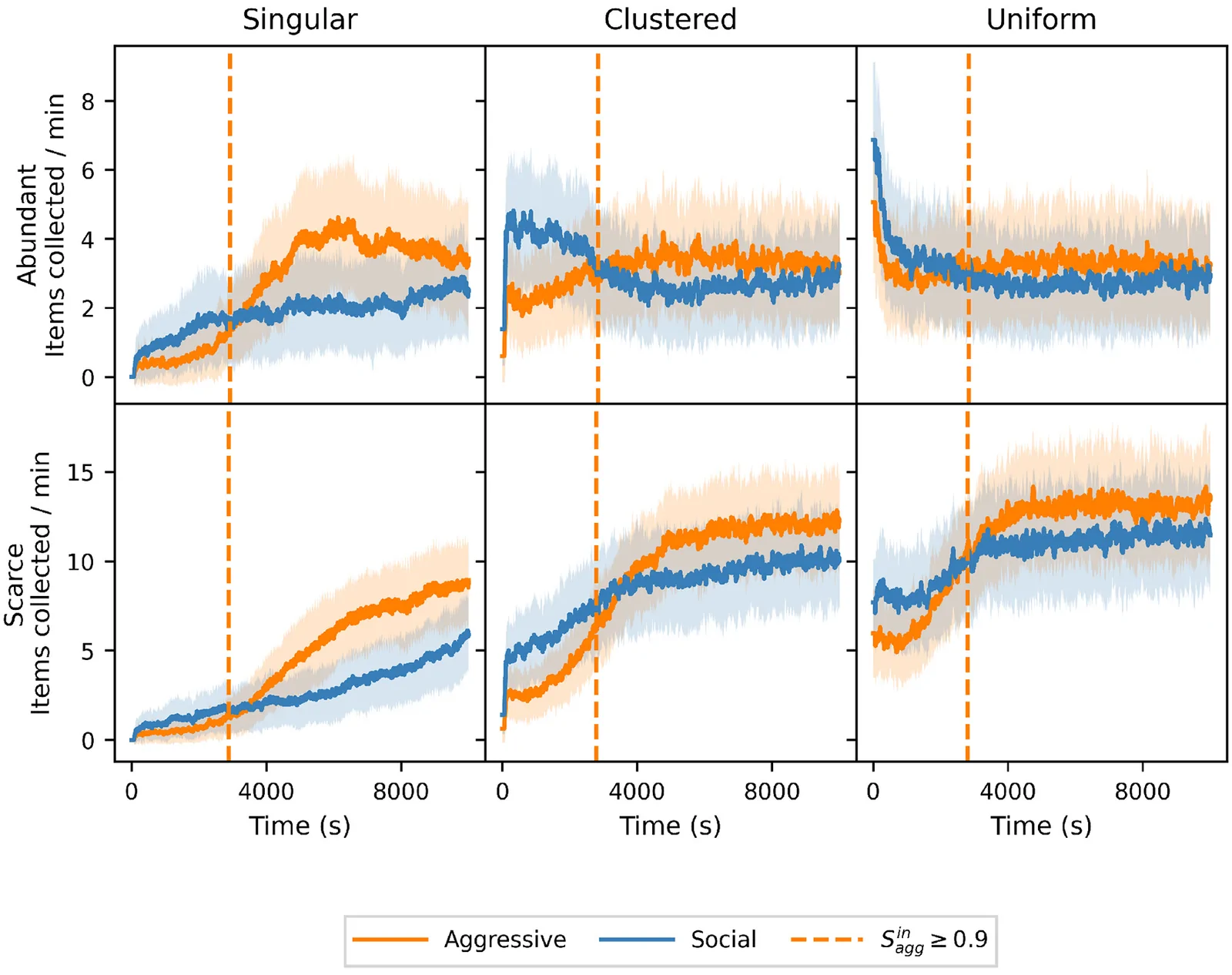
Evolution of the rate of collected resource items over time (average per nest) for different item distributions (columns), item availability (rows) and robot species (colors). The sub-groups have a different initial sociality. The dashed orange lines indicate the time at which aggressive agents (initial sociality = 0.1) reach an in-group sociality of 0.9. Lines show mean values across 100 simulation runs, and shaded areas indicate (±1) standard deviation.

Figure 9 shows the average rate of item collection per nest. In the abundant environments, the social species is initially more successful at resource collection, as high initial sociality enables earlier exploration (cf. heterogeneous panels of Figure 2). Indeed, aggressive robots trigger rejections more often than social robots, and therefore return to their nest earlier and more often. This benefit is, however, limited to cases where items are relatively easy to find (i.e., when there is no singular distribution). Notably, the collection rate of aggressive agents obeys a transition function, with a pivot at the moment when in-group cooperation is reached (i.e., when sociality among the aggressive species becomes greater than 0.9). Before this point, aggressive agents perform worse than social agents as they are unable to explore due to their failed interactions, and are forced to return to their nest. Yet once aggressive robots converge vocabulary-wise, they become as able to explore and collect food as the social agents—but since they are still aggressive toward outsiders, this triggers more rejection (and thus return to the nest) of the social robots. Therefore aggressive robots are able to *enforce* a foraging territory that does not have to be shared.

This dynamic is especially prevalent in the scarce-singular case, where the scarcity of resources favors aggressive agents even more seeing as the resources are easier to guard, which in turn stops social agents from foraging efficiently.

## 4 Discussion

For sociality to play a role in the emergence of shared communication, reduced aggression or increased tolerance must provide some functional advantage. Here we measured said advantage over a foraging task, coupled with the self-organization of a simple language (which serves as a model of social agreement). This language, in turn, determines the behavior of robots toward each other: They will be more likely to attack robots they have failed to communicate with in the past, rather than engaging in the cultural evolution of the language. We modeled a scenario with two sub-groups of robots, inhabiting separate nests and tested the impact of aggressive and social behaviors in different environmental scenarios to test when and how it might be advantageous for robots' survival. The insights provided by our model are presented, in terms of foraging efficiency as a proxy for survival efficiency on the short and long term, as a visual summary in Table 2.

**Table 2:** Visual summary of foraging efficiency on short and long terms.

Renewal	Scenario	Singular		Clustered		Uniform	
Abundant	Homogeneous						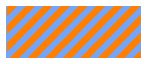
	Heterogeneous	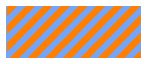					
Scarce	Homogeneous					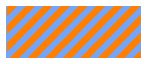	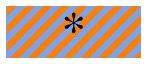
	Heterogeneous	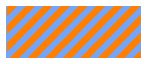			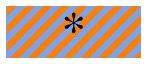		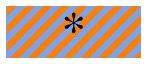
	Term	Short	Long	Short	Long	Short	Long

Blue cells indicate scenarios in which social robots foraged more resources than aggressive robots, while orange cells indicate scenarios in which aggressive robots foraged more resources than social robots, for a given term, scenario, and environmental condition. Hatched cells indicate that no clear advantage was observed for either species. Asterisks indicate cases where the environment was over exploited, thus preventing the sub-groups from achieving better foraging performances. Statistical inference of this table is based on the linear mixed-effects model shown in Figure 2.

Our results show that in-group bias [the documented propensity (Petersen et al., 2004) to favor individuals from one's own group over others] naturally emerges in aggressive agents due to greater initial exposure to their in-group, without necessarily requiring any ability to *a priori* distinguish in-group from out-group agents (in contrast, social agents display little bias as they are open to all interactions from the start). Indeed, we show that an in-group bias emerges from the foraging behavior itself, as well as from the ecological niche: extensive food availability promotes exploitation of known resources close to the nest and therefore familiarization with strangers. These two factors increase linguistic convergence locally, which acts as a marker of nest belonging and promotes an in-group bias. This result is consistent with previous studies that showed that groups with high in-group interactions display a stronger in-group bias than isolated individuals (Petersen et al., 2004), and that the in-group bias is, at least partly, nurtured (Skinner and Meltzoff, 2019), and indicates that despite the relative simplicity of our model, it is able to predict realistic emergent features found in many social animals, including humans.

Moreover, in heterogeneous environments where one nest is inhabited by aggressive agents and the other by social agents, the in-group bias is not as marked, and quickly reduces with out-group interactions. As social agents start with a high propensity toward playing language games, they continuously interact with aggressive agents until they can establish a social consensus with them. Once a social consensus is established, any interaction allowed by an aggressive agent is positive, which rapidly erodes the in-group bias. This phenomenon would ultimately favor domestication as it provides a “taming” mechanism, i.e., the more social species is able to render another species less aggressive by offering only positive interactions. Let us note that this process is only possible because the agents' sociality only decreases when the MNG fails. Indeed, in cases where the sociality is initially low, which results in an attack, the relationship between the two agents is not impacted. If attacks decreased sociality, the high level of initial interaction described above would only result in lowering the sociality, thus preventing any form of taming, or even in-group sociality for the aggressive species.

Once we validated our model's ability to self-organize an in-group bias, we tested which environmental conditions would promote lower aggression and what effect this may have on robots' communication dynamics. Specifically, we explore two types of environmental conditions that have been implicated in the self-domestication literature as potential triggers for selection against aggression in humans in the middle and late Paleolithic (Pisor and Surbeck, 2019; Spikins et al., 2021) as well as in other species that have been hypothesized to have gone through a self-domestication process (Hare et al., 2012; Raviv et al., 2023).

The first scenario is an abundant environment in which food supply is rich and reliable and the risk of aggression is relatively low (Pisor and Surbeck, 2019; Hare et al., 2012). Such safe and abundant environments are considered the most prominent trigger for a relaxation of selection pressures in domesticated animals [i.e., humans provide animals with a food-abundant niche (Hare, 2017)], as well as in bonobos (Hare et al., 2012) and elephants (Raviv et al., 2023) which have also been identified as self-domesticated. We could observe a similar dynamic in our simulations, but only in the absence of aggressive agents—as the latter would enforce territories that impede the resource collection of social agents. In contrast, when facing agents with a similar degree of high sociality, social agents performed much more efficiently than aggressive agents, as they were able to exploit the environment without useless impediments. Under the stable and abundant resource conditions modeled here, territorial exclusion does not provide an immediate foraging advantage.

The second scenario suggests that it is rather harsh environmental conditions that promoted tolerance and friendly behavior in humans (Spikins et al., 2021; Pisor and Jones, 2021). Specifically, it was suggested that climate deterioration during the last glaciation resulted in scarcer environments with fewer resources and/or with large spatial and temporal fluctuations in resource availability, which forced humans to rely on non-local food sources as means of managing the risks of resource shortfalls (Pisor and Surbeck, 2019). These changes in foraging ecology may have required humans to develop better between-community relationships in order to ensure access to these non-locally-available resources, as exchanging and sharing food between sub-groups became beneficial for survival (Spielmann, 1986). Our results, however, do not support this scenario: we found that in scarce environments, sociality is only advantageous in the short-term. Indeed, social agents initially enable their group members to explore the environment as they are less likely to attack them than aggressive agents. However, in the long term, aggressive agents develop a stronger in-group bias, enabling their group members to freely explore, while protecting resources from other groups.

In any case, our results indicate that regardless of the specific environmental setup, sociality is clearly beneficial at the beginning of the experiment. Less-social agents have to overcome their innate aggression toward one another before organizing their territories, thus wasting crucial time. Meanwhile, more social agents are able to collect most of the available resources before they could be protected. This might echo the founder effects—a theory that suggests a prosocial advantage in the first exploration into a new area (Brooks and Yamamoto, 2021). Indeed, an entire population presented at once with a new environment (as is the case in our setup) faces a unique set of challenges as the position of resources and competing species is unknown, resulting in a lack of clear territories. At the same time, this as-of-yet underexploited environment presents an abundance of resources.

Our results are consistent with a recent network-based iterated model (Sains et al., 2023) that found that networks of smaller communities are less prone to developing a single language than more evenly connected networks. Individuals communicating with communities having different languages indeed make these multiple languages compete within their own communities, which therefore acts as a frontier and prevents the whole population from reaching a full consensus. In the future, we are interested in developing our model to accommodate more than two populations, and verify whether these results apply to our model too. A previous work on multi-agent simulations (Szilágyi et al., 2023) presented a detailed model of the confrontational scavenging scenario, in a shared Euclidean space. This scenario, which takes place on a much longer timescale than the one studied in the present work, states that language development, enabled by an abundant environment (as hinted by the present study), would have allowed early humans to survive a dietary shift as rich arboreal environments gave way to savannas. The model in Szilágyi et al. (2023), however, is concerned with relatively advanced communication enabling agents to communicate fairly accurate information about the distance and risk associated with a prize. By comparison, our work focuses on the inception of language, and the social behavior enabling it. This is the very first stage of language development under the self-domestication hypothesis, with “rudimentary language, possibly dominated by the expressions of emotions through single-word commands, threats, and exclamations” enabled by a reduction in reactive aggression (Beńıtez-Burraco and Progovac, 2020). Specifically, our model does not use the evolved language for the purpose of foraging (i.e., the vocabulary does not correspond to actual objects in the environments), but only as a sign of recognition between agents.

We see our study as a first step toward modeling and understanding the potential effects of self-domestication on language formation, and therefore aim to tackle the next steps of language development, by enabling symbolic communication, more advanced grammars, and proactive aggression. In future work, we plan to add emergent meaning to our simulation by associating words with experiences of the real world. Since different robots experience the environment from different points of view, the sensory data they associate with a shared word is usually not exactly the same.

As well as the progress we presented in the field of evolutionary linguistics, we introduced a novel modification to classic swarm robotics models (namely, individual sociality and relationships, instead of homogeneous interactions) that is also suitable for swarm robotics applications. Specifically, swarm intelligence studies tend to assume simplistic connectivity patterns such as interacting uniformly with agents within a given range. However, collective behaviors might be subject to more complex social networks, with individual connection strength (Rosenthal et al., 2015). In the same way that edge weights in neural networks enable powerful decision-making, individual connection strength between agents might do the same in robotic swarms. On a more general level, studying the interplay between cultural evolution dynamics, the self-organization of languages, and swarm behaviors can greatly benefit the understanding of all these phenomena (Cambier et al., 2020; Whiten et al., 2022; Coucke et al., 2023).

## Data Availability

The raw data supporting the conclusions of this article will be made available by the authors, without undue reservation.

## Footnotes

1 On average, fewer than one robot reached the arena boundary per 100 simulations, with no measurable effect on the overall dynamics.
