# Supplementary material for: Pros and cons of sociality across ecological niches: a swarm robotics approach
Source: Front Psychol. 2026 Sep 8;17:1872416. doi: 10.3389/fpsyg.2026.1872416 (PMC13593817; doi:10.3389/fpsyg.2026.1872416)
Supplement: Supplementary file 1 [file Data_Sheet_1.pdf]

Supplementary Material

1 SUPPLEMENTARY TABLES AND FIGURES

1.1 Linear mixed-effects model of item collection

Term	Estimate	SE	t-value
Intercept	210.163	1.929	108.932
Type	230.760	2.668	86.491
Spread: Singular vs. Clustered	174.360	4.621	37.731
Spread: Singular vs. Uniform	354.355	4.621	76.681
Term	632.407	2.668	237.032
Heterogeneity	46.010	3.268	14.080
Environment	-78.438	2.668	-29.399
Type × Spread: Singular vs. Clustered	132.540	6.535	20.281
Type × Spread: Singular vs. Uniform	108.150	6.535	16.549
Type × Term	174.153	3.773	46.156
Spread: Singular vs. Clustered × Term	603.675	6.535	92.372
Spread: Singular vs. Uniform × Term	716.795	6.535	109.680
Type × Heterogeneity	-163.123	4.621	-35.299
Spread: Singular vs. Clustered × Heterogeneity	29.880	8.004	3.733
Spread: Singular vs. Uniform × Heterogeneity	60.645	8.004	7.577
Term × Heterogeneity	218.707	4.621	47.327
Type × Environment	-188.050	3.773	-49.839
Spread: Singular vs. Clustered × Environment	-41.375	6.535	-6.331
Spread: Singular vs. Uniform × Environment	-194.180	6.535	-29.713
Term × Environment	-478.330	3.773	-126.772
Heterogeneity × Environment	-45.975	4.621	-9.949
Type × Spread: Singular vs. Clustered × Term	-622.130	9.242	-67.313
Type × Spread: Singular vs. Uniform × Term	-892.605	9.242	-96.578
Type × Spread: Singular vs. Clustered × Heterogeneity	-45.740	11.319	-4.041
Type × Spread: Singular vs. Uniform × Heterogeneity	-77.120	11.319	-6.813
Type × Term × Heterogeneity	-482.110	6.535	-73.770
Spread: Singular vs. Clustered × Term × Heterogeneity	-256.595	11.319	-22.669
Spread: Singular vs. Uniform × Term × Heterogeneity	-408.185	11.319	-36.061
Type × Spread: Singular vs. Clustered × Environment	-206.895	9.242	-22.386
Type × Spread: Singular vs. Uniform × Environment	-207.585	9.242	-22.460
Type × Term × Environment	-180.435	5.336	-33.814
Spread: Singular vs. Clustered × Term × Environment	-618.905	9.242	-66.965
Spread: Singular vs. Uniform × Term × Environment	-760.705	9.242	-82.307
Type × Heterogeneity × Environment	158.587	6.535	24.266
Spread: Singular vs. Clustered × Heterogeneity × Environment	-59.895	11.319	-5.291
Spread: Singular vs. Uniform × Heterogeneity × Environment	-86.940	11.319	-7.681

Continued on next page

Table S1 – continued from previous page

**Term**	**Estimate**	**SE**	***t*-value**
Term × Heterogeneity × Environment	-136.277	6.535	-20.852
Type × Spread: Singular vs. Clustered × Term × Heterogeneity	643.470	16.008	40.197
Type × Spread: Singular vs. Uniform × Term × Heterogeneity	989.805	16.008	61.831
Type × Spread: Singular vs. Clustered × Term × Environment	581.840	13.071	44.515
Type × Spread: Singular vs. Uniform × Term × Environment	879.100	13.071	67.258
Type × Spread: Singular vs. Clustered × Heterogeneity × Environment	158.885	16.008	9.925
Type × Spread: Singular vs. Uniform × Heterogeneity × Environment	169.155	16.008	10.567
Type × Term × Heterogeneity × Environment	350.022	9.242	37.872
Spread: Singular vs. Clustered × Term × Heterogeneity × Environment	141.745	16.008	8.855
Spread: Singular vs. Uniform × Term × Heterogeneity × Environment	279.045	16.008	17.431
Type × Spread: Singular vs. Clustered × Term × Heterogeneity × Environment	-558.650	22.639	-24.677
Type × Spread: Singular vs. Uniform × Term × Heterogeneity × Environment	-864.170	22.639	-38.172

Table S1: Fixed-effect coefficients of the linear mixed-effects model of item collection. The model predicts the cumulative number of items collected per scenario from robot sociality, resource spread, time period, population composition, and resource availability, including all interactions between these predictors and a random intercept for simulation seed. Short and long terms respectively refer to the periods before and after aggressive agents, with an initial sociality of 0.1, reach an in-group sociality of 0.9 in the corresponding experimental condition. Estimates, standard errors, and t-values are reported.

1.2 Figures

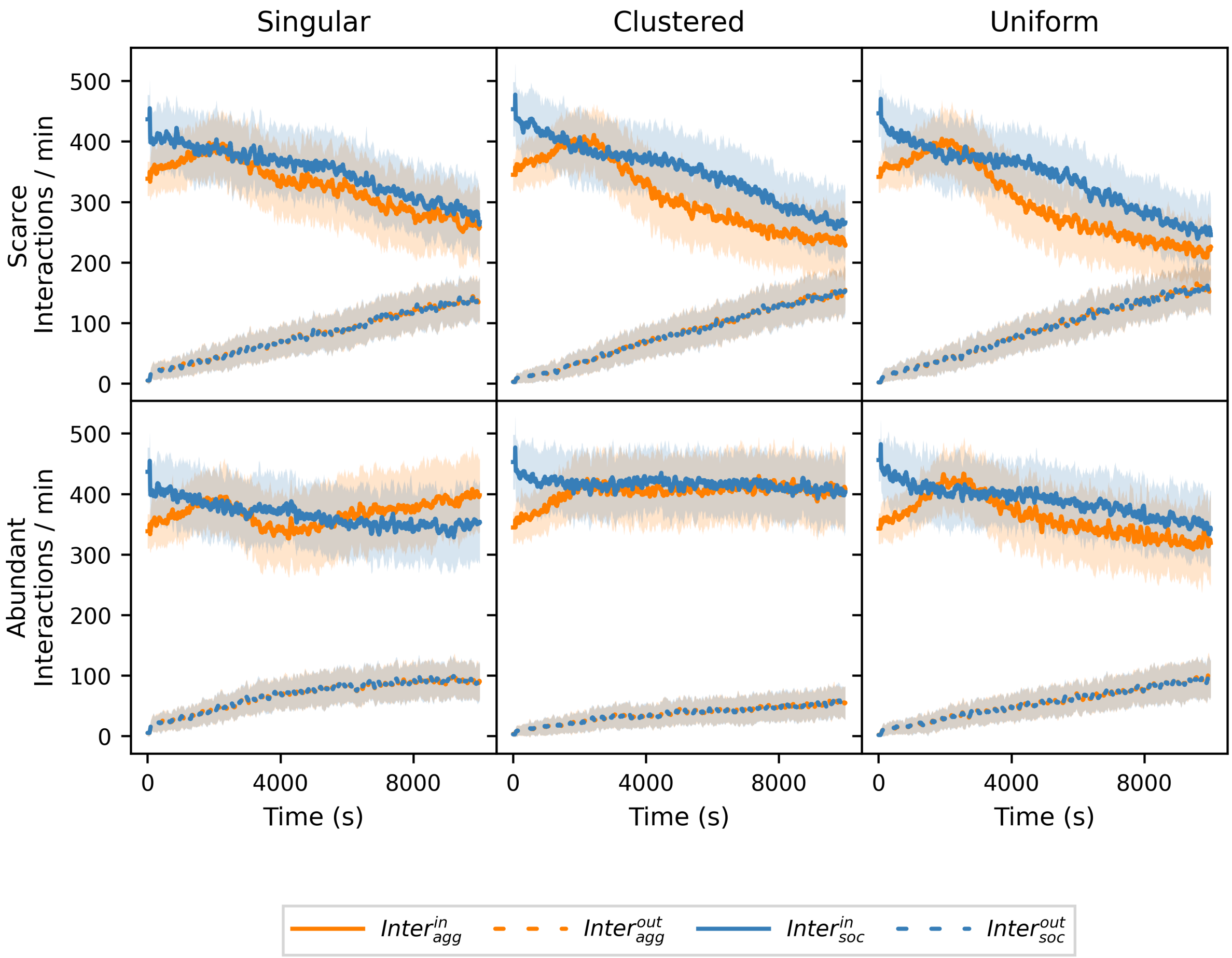

Figure S1. Evolution of the total rate of interactions between agents over time for different item distributions (columns), item availability (rows) and initial sociality (colors), in the heterogeneous case (each sub-groups has a different initial sociality). Lines show mean values across 100 simulation runs, and shaded areas indicate ± 1 standard deviation. Subscripts *agg* and *soc* refer to aggressive and social robots, respectively, whereas superscripts *in* and *out* refer to interactions with in-group robots and out-group robots, respectively.

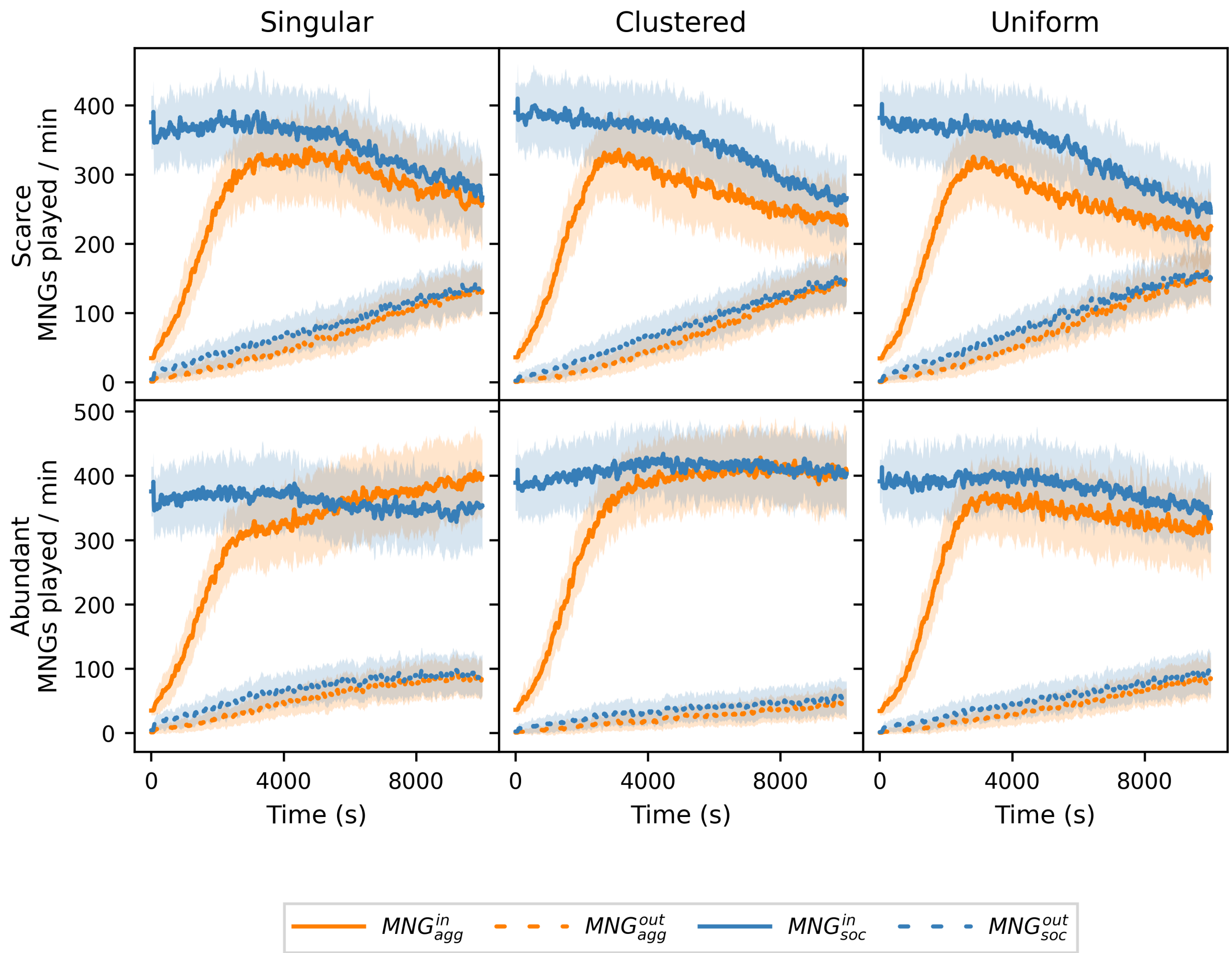

Figure S2. Evolution of the total rate of naming games played over time for different item distributions (columns), item availability (rows) and initial sociality (colors), in the heterogeneous case (each sub-groups has a different initial sociality). Lines show mean values across 100 simulation runs, and shaded areas indicate ± 1 standard deviation. Subscripts *agg* and *soc* refer to aggressive and social robots, respectively, whereas superscripts *in* and *out* refer to interactions with in-group robots and out-group robots, respectively.

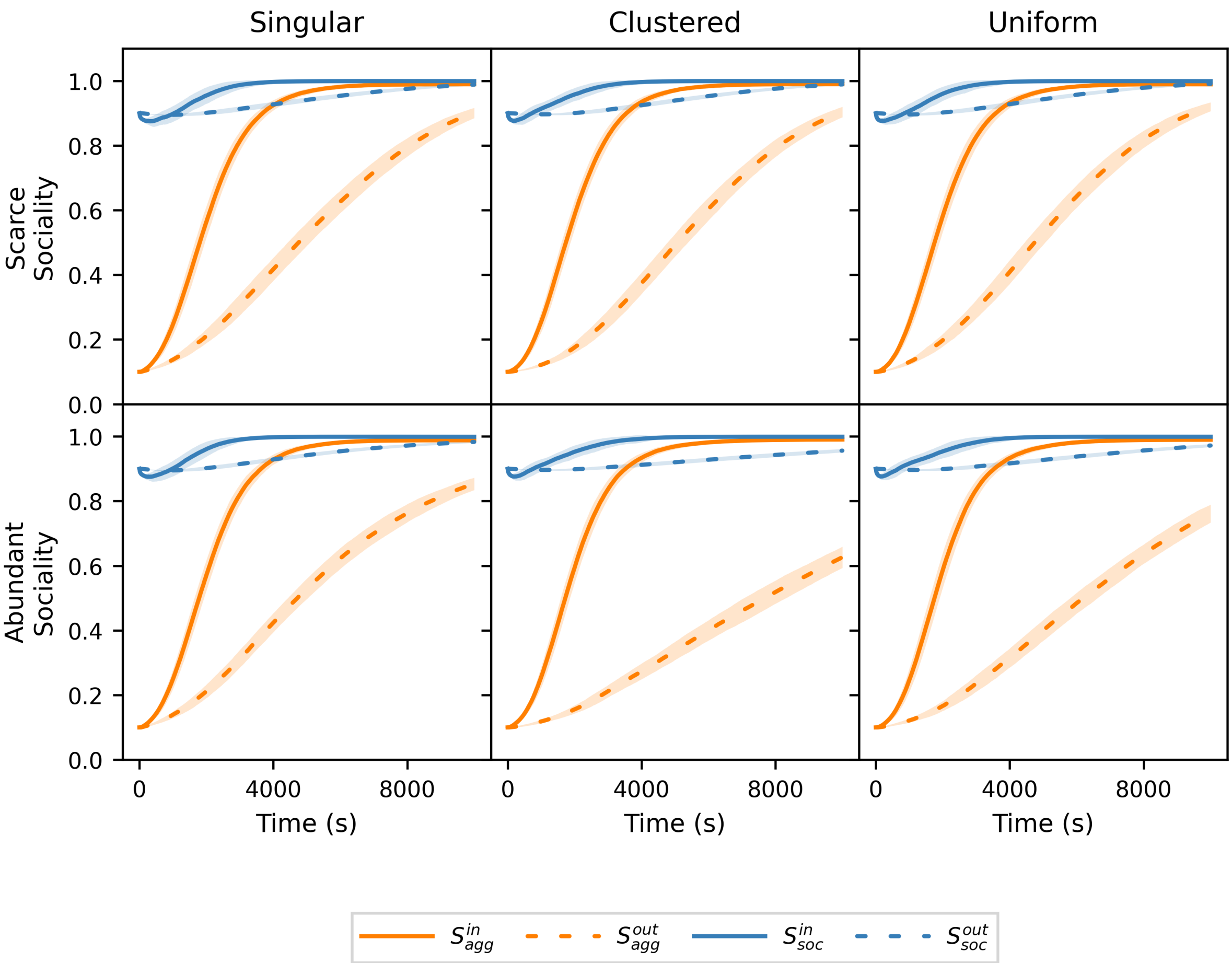

Figure S3. Evolution of the sociality over time for different item distributions (columns), item availability (rows) and initial sociality (colors), in the heterogeneous case (each sub-groups has a different initial sociality). Lines show mean values across 100 simulation runs, and shaded areas indicate ± 1 standard deviation. Subscripts *agg* and *soc* refer to aggressive and social robots, respectively, whereas superscripts *in* and *out* refer to interactions with in-group robots and out-group robots, respectively.
